# Smart Dual-Functionalized Gold Nanoclusters for Spatio-Temporally Controlled Delivery of Combined Chemo- and Photodynamic Therapy

**DOI:** 10.3390/nano10122474

**Published:** 2020-12-10

**Authors:** Andrea Tabero, Oriol Planas, Thibault Gallavardin, Ingrid Nieves, Santi Nonell, Angeles Villanueva

**Affiliations:** 1 Departamento de Biología, Universidad Autónoma de Madrid, Darwin 2, 28049 Madrid, Spain; andrea.tabero@uam.es; 2 Institut Químic de Sarrià, Universitat Ramon Llull, 08017 Barcelona, Spain; oriolplanasm@gmail.com (O.P.); thibault.gallavardin@univ-rouen.fr (T.G.); ingrid.nieves.ca@gmail.com (I.N.); 3 Instituto Madrileño Estudios Avanzados IMDEA Nanociencia, C Faraday 9, 28049 Madrid, Spain

**Keywords:** photodynamic therapy, chemophototherapy, gold nanoclusters, photonanomedicine, doxorubicin, protoporphyrin IX

## Abstract

We report the preparation of gold nanoclusters (AuNCs) as a delivery vehicle for the clinically approved photodynamic and chemotherapeutic agents Protoporphyrin IX (PpIX) and doxorubicin (DOX), respectively, and their effect on tumor cells. DOX was attached to the gold nanoclusters through a singlet oxygen-cleavable linker and was therefore released after PpIX irradiation with red light, contributing, synergistically with singlet oxygen, to induce cell death. The doubly functionalized AuNCs proved more effective than a combination of individually functionalized AuNCs. Unlike free DOX, the photoactive nanosystem was non-toxic in the absence of light, which paves the way to introduce a spatiotemporal control of the anticancer therapy and could contribute to reducing the undesirable side effects of DOX.

## 1. Introduction

Nanomedicines have become very successful alternatives in very different clinical areas, namely diagnosis (nanodiagnosis), drug delivery (nanotherapy), and regenerative medicine [1,2]. Currently, there are more than 50 products in clinical use obtained from this technology and more than 200 clinical trials (completed and ongoing) in infectious, ophthalmological, dental, and oncological diseases, among others [3,4].

Drug delivery techniques based on nanotechnology can modify drug solubility or diffusivity and to enhance biological availability through the enhanced permeability and retention (EPR) effect. However, the most important advantage of nanoparticles as drug delivery systems is the co-delivery of multiple therapeutic agents on the same platform which allows a spatio-temporal controlled release of attached drugs [4,5]. Out of this innovative approach emerged the concept of photonanomedicine, which develops light-activable nanosystems that integrate therapeutics and diagnostics (theranostics), selection of the molecular agents and perfect control of the time, the place and the dosage when releasing therapeutic agents [3]. Photonanomedicine is based on the conjunction of nanotechnology and photodynamic therapy (PDT).

The light-based cancer therapy known as PDT involves the administration of a photosensitizing drug (PS) that accumulates mainly in tumor cells. The PS is not cytotoxic per se, but upon activation with light of specific wavelengths triggers the formation of reactive oxygen species (ROS), mostly singlet oxygen (^1^O_2_) that lead to irremediable cell damage, and consequently tumor regression [6].

PDT was approved 25 years ago to treat certain tumors and its application has spread quickly to other areas of medicine such as ophthalmology [7,8], dermatology [9,10,11], infectious diseases [12], and cardiology [13]. However, undoubtedly, the largest area of application of PDT is oncology, where it has been approved for the treatment of various types of cancers including head and neck tumors, basal-cell carcinoma, cervical, endobronchial, esophageal, bladder, and gastric cancer [14]. PDT is highly selective as it needs the presence of three elements simultaneously: a photosensitizer (PS), molecular oxygen and light. These elements are not toxic by themselves, so it is possible to avoid general toxicity by using light only in the desired area [15].

However, PDT has some limitations. The nature of the PS is critical for its efficacy. In addition to having adequate photochemical properties, the PS should be soluble in biological media, highly selective for the tumor, and quickly removed from the human body [16]. It is challenging to convey all these features into a single small molecule, so the current trend is to develop PSs attached to targeting moieties, e.g., antibodies, or incorporated into different vehicles such as nanoparticles.

The potential of PDT for overcoming drug resistance or survival pathways is currently being actively explored. New data indicate that the way PDT produce damage in cancer cells and their microenvironment can be used to avoid cancer drug resistance, to minimize the initiation of survival pathways and, in some cases, even to reconvert resistant cells and make them sensible to standard therapies [17,18,19,20].

For this reason, the use of combinations of various therapeutic modalities with non-overlapping toxicities in a unique nanosystem is being introduced to increase the efficacy of PDT [21,22,23,24]. Here, the concept of chemophototherapy arises as to the combination of PDT and conventional chemotherapy with the potential to provide the capacity to implement a more effective therapy for neoplastic diseases with reduced side effects [25].

Because of their specific characteristics, gold nanomaterials are under extensive investigation for many biomedical approaches and therapies. It is possible to synthesize them in different forms and dimensions, they can be easily functionalized by all kinds of biomolecules due to the presence of negative charge on its surface, they are biocompatible and non-toxic and can provide greater stability to loadings on their surfaces [26,27]. Depending on their size and shape, metallic nanomaterials can be classified as nanoclusters, whose diameter is lower than 2 nm, and nanoparticles (or nanocrystals), with a diameter larger than 5 nm. Nanoclusters (MNCs) can be considered to be groups of up to a few hundreds of metallic atoms surrounded by a stabilizing agent. Their size is insufficient to support a superficial localized plasmon. Nonetheless, MNCs possess unique features that make them worth studying [28]. Gold nanoclusters, bridging the gap between individual atoms and large nanocrystals, are the smallest-available nanoparticles that can be used as drug carriers, to enhance the solubility of the drugs, and keep them sufficiently close for a pre-designed purpose, as reported herein. Compared to the larger gold nanoparticles, nanoclusters reduce drastically the amount of gold internalized by cells and therefore decrease the possibility of toxic effects. Por the specific case of hydrophobic porphyrin photodynamic photosensitizers, the use of gold nanoclusters as carriers precludes aggregation of the porphyrin and the concomitant deterioration of its photosensitizing ability.

Here, we used gold nanoclusters (AuNCs) to combine PDT and chemotherapy in a light-activated control system. To evaluate the therapeutic potential of this system, we have selected two already clinically approved drugs: Protoporphyrin IX (PpIX) as PS and the common chemotherapeutic agent Doxorubicin (DOX). By using smart chemical design, DOX was attached to alkyl-thiol tetraethyleneglycol stabilized gold nanoclusters through a ^1^O_2_-cleavable link [29]. Thus, DOX was only released after PpIX irradiation, which could contribute to reducing undesirable side effects.

## 2. Materials and Methods

### 2.1. Cell Cultures

HeLa cells (ATCC^®^ CCL-2^TM^, LGC Standards S.L.U., Manassas, VA, USA) were cultured in DMEM (Dulbecco’s modified Eagle’s medium) supplemented with 10% FBS, 50 U·mL^−1^ penicillin, and 50 μg·mL^−1^ streptomycin (Thermo Fisher Scientific, Waltham, MA, USA). The culture solution was sterilized by filtration (pore size 0.22 µm) (Merck Millipore, Billerica, MA, USA). Following ATCC recommendations, cells were cultured at 37 °C and 5% CO_2_ atmosphere. Subconfluent cell cultures were used for experiments.

### 2.2. Drugs and Nanoconjugates

Gold nanoclusters were prepared by reduction of tetrachloroauric acid in the presence of stabilizing ligands (see Supplementary Information). Protoporphyrin IX (PpIX) and Doxorubicin (DOX) were purchased from Frontier Scientific (Logan, UT, USA) and Sigma Aldrich, Chemical Co. (St. Louis, MO, USA), respectively. The protocols for conjugation to the nanoclusters and preparation of the corresponding linkers are described in the Supplementary Materials. Stock solutions were prepared in dimethyl sulfoxide (DMSO) and stored at 4 °C until use. Before incubation, stock solutions were allowed to equilibrate with room temperature for 30 min and were subsequently sonicated for 15 min to ensure complete dispersion of the nanoconjugates. Table 1 compiles information about gold and drug concentration in stock solutions.

After sonication, stock solutions were diluted in culture medium to the desired concentration (2.3 µM and 1.4 µM for PpIX and DOX, respectively). Unsubstituted AuNCs were used to assess the intrinsic toxicity of these nanoparticles.

### 2.3. Photodynamic Treatments

Subconfluent cultures of HeLa cells were incubated for 18 h with different nanoconjugates, washed three times in DMEM, and irradiated. During irradiation and post-treatment time, cells were maintained in culture medium. Four different treatments were applied: single treatments with either AuNC-DOX or AuNC-PpIX, combined unimodal treatments with equal concentrations of AuNC-DOX and AuNC-PpIX, and combined bimodal treatments with the binary nanoconjugate AuNC-DOX-PpIX. In all these treatments, DOX and PpIX concentrations were maintained constant (DOX 1.4 µM, PpIX 2.3 µM). Irradiations were performed with a LED emitting red light (λ = 632 ± 10 nm) (LED Par 64 Short; Showtec, Burgebrach, Holland). Fluence rate [mW·cm^−2^] was measured with the PM100A handheld optical power meter (Thorlabs, Newton, NJ, USA) and irradiation time (5.6 min) was adjusted for a total light dosage of 2.4 J·cm^−2^ with a beam spot diameter of 15 cm. In parallel with these experiments, cells incubated with nanoconjugates and free PpIX and DOX were maintained in dark conditions to measure possible cellular toxicity exerted by compounds themselves. Similar experiments were carried out to discard toxicity of irradiation in the absence of compounds and DMSO present in compound solutions (DMSO concentration up to 0.2%) (data not shown).

### 2.4. 3-(4,5-Dimethylthiazol-2-yl)-2,5-Diphenyltetrazolium Bromide (MTT) Assay

Cytotoxicity of treatments was measured by MTT cell viability assay 48 h after each treatment. Cells were incubated with a 50 μg·mL^−1^ solution of MTT (Sigma Aldrich, Saint Louis, MO, USA) in culture medium for 3 h. Reduced formazan was dissolved in DMSO and absorbance was measured at 542 nm in a SpectraFluor spectrophotometer (Tecan Group Ltd., Mannedorf, Switzerland). Cell survival was determined as the percentage of absorption of treated cells compared to that of the control cells. Statistical significance was calculated by one-way ANOVA analysis with pairwise comparisons between means. Three independent experiments were performed for each condition.

### 2.5. Neutral Red (NR) and Hoechst-33258 (H-33258) Staining

Neutral Red (Panreac, Barcelona, Spain; 0.5% in distilled water, 2 min) and H-33258 (Sigma Aldrich; 5 μg.mL^−1^ in distilled water, 3 min) staining were performed to assess morphological and nuclear alterations (respectively) in HeLa cells 48 h after treatments. Prior to incubation with NR and H-33258 cells were fixed with methanol at −20 °C for 5 min. After staining, preparations of cells were air-dried, mounted in DePeX (Serva, Heidelberg, Germany) and observed under light and fluorescence microscopy.

### 2.6. Subcellular Localization

Confocal microscopy was used to evaluate the incorporation of compounds into HeLa cells. After 18 h of incubation with 1.4 μM of DOX or AuNCs-DOX, or 2.3 μM of PpIX or AuNC-PpIX, cells were washed and maintained in culture medium during visualization under confocal fluorescence microscopy.

### 2.7. Cellular Uptake Analysis by Flow Cytometry

Cells were incubated with 1.4 μM of DOX/AuNCs-DOX or 2.3 μM of PpIX/AuNC-PpIX for 18 h. Just after washing or 1 h later, cells were trypsinized and centrifuged (4 min, 1200 r.p.m., rotor radius: 7 cm). After discarding the supernatant, the pellet was resuspended in PBS, and analyzed by flow cytometry. Fluorescence of internalized compounds was analyzed using a Coulter Epics XL-MCL flow cytometer (Beckman-Coulter Inc., Fullerton, CA, USA) with an argon laser line (488 nm) and 695/30 nm filter for PpIX and 620 BP filter for DOX. Cells incubated with empty AuNCs were also measured to discard background signal associated with AuNCs.

### 2.8. ƴ-H2AX Indirect Immunofluorescence Assay

DNA damage after treatments was examined by immunofluorescence detection of histone ƴ-H2AX. Cells were fixed in formol-PBS (1:10) for 20 min at 4 °C, washed (PBS, 3 × 5 min), and treated 5 min with 0.5% Triton X-100 for cell membrane permeabilization. Cells were then incubated 30 min in blocking solution (5% bovine serum albumin, 5% FBS, 0.02% Triton X-100 in PBS), followed by 1 h incubation at 37 °C with primary antibody (1:400, monoclonal mouse phospho-histone H2AX (Ser140); Thermo Fisher Scientific). After washing, cells were incubated with secondary antibody (1:100 Fab specific goat anti-mouse AlexaFluor 488; Thermo Fisher Scientific) and, finally, counterstained using H-33258 for nuclei visualization and mounted with ProLong Gold antifade reagent. Fiji ImageJ software [30] was used to calculate the percentage of positive cells and relative ƴ-H2AX expression after each treatment.

### 2.9. Transmission Electron Microscopy

Cell death and cellular uptake mechanism were studied by transmission electron microscopy 48 h after irradiation of just after washing (for cell death and uptake mechanism visualization, respectively). A solution of 2% glutaraldehyde + 1% tannic acid in 0.4 M HEPES buffer at pH 7.2 was used for cell fixation (2 h at room temperature). Then, cells were postfixed for 1 h in PBS with 1% osmium tetroxide and 0.8% potassium ferricyanide (Taab Laboratories, England, UK), dehydrated and embedded in Epon. Uranyl acetate and lead citrate were used for double staining of ultrathin sections of samples. Visualization was performed using a JEOL (Tokyo, Japan) JEM-1011 transmission electron microscope complemented with a Gatan Erlangshen ES 1000W camera (Pleasanton, CA, USA).

### 2.10. Western Blot for Cleaved Caspase-3

Analysis of protein expression alterations after treatments was performed by western blot techniques. 18 h after treatments, control, and treated cells with either AuNC-DOX or AuNC-PpIX-PDT or the mixture AuNC-DOX + AuNC-PpIX (Combined Unimodal), or AuNC-DOX-PpIX (Combined Bimodal) were processed. Cells were incubated with RIPA buffer (Santa Cruz Biotechnology, Dallas, TX, USA) to extract whole protein content. After determination of protein concentration with BCA assay kit (Pierce, Rockford, IL, USA), samples were separated on 12% SDS-PAGE and transferred to an Immobilon-P PVDF Membrane (0.45 µm pore size) (Merk Millipore) at 280 mA for 2 h. Overnight incubation at 4 °C with rabbit monoclonal anti-cleaved-caspase 3 antibody (Cell Signaling, Danvers, MA, USA), and mouse monoclonal anti-α-Tubulin antibody (Sigma Aldrich), followed by incubation with secondary antibody (sheep anti-rabbit and sheep anti-mouse IgG conjugated to horseradish peroxidase; Agilent, CA, USA) was used for chemiluminescent detection of proteins in PVDF membrane. Bands were developed using ECL Clarity and chemiluminescence detection system ChemiDoc XRS+ (Bio-Rad, Hercules, CA, USA).

### 2.11. Optical Microscopy

Analysis of subcellular localization of drugs was performed with a multispectral Leica (Wetzlar, Germany) TCS SP8 confocal microscope operating with 405 nm (argon–UV) and WLL2 (White Light Laser) laser lines. ƴ-H2AX indirect immunofluorescence was visualized with an Olympus (Tokyo, Japan) BX63 epifluorescence microscope equipped with a CoolLED’s pE-300 light source and Olympus DP74 camera. During all the experiments, cell growth and morphology were monitored with and Leica DMIL LED inverted microscope equipped with a Leica DFC 420c camera. In addition, time-lapse video microscopy of living cells after combined treatment was carried out with a Leica DMI6000B microscope with an incubation system and an OrcaR2 monochrome digital camera for image detection (Hamamatsu Photonics, Shizuoka, Japan).

## 3. Results and Discussion

### 3.1. Synthesis and Characterization of the AuNCs

**Figure 1:**
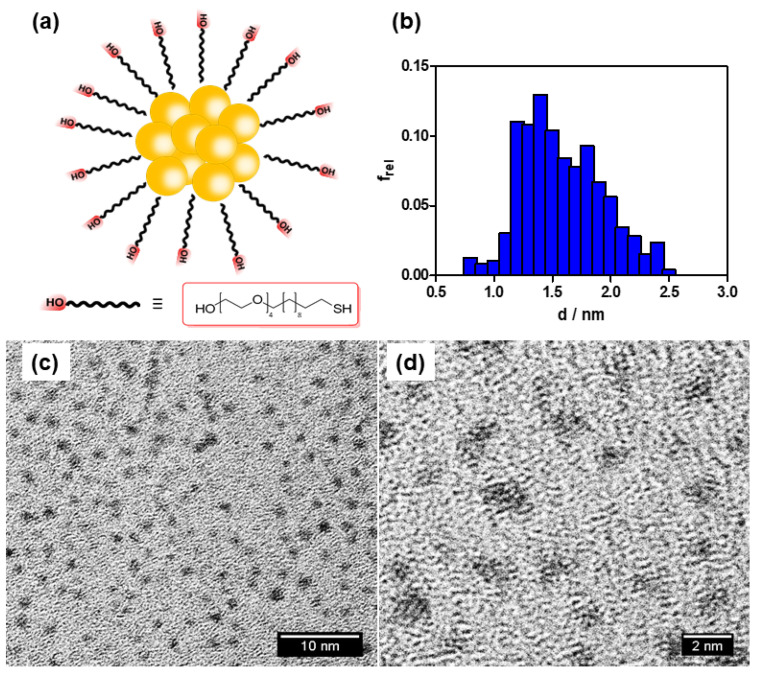
Characterization of gold nanoclusters. (**a**) Schematic representation of the structure of AuNCs; (**b**) Particle size distribution; (**c**) TEM micrography at 120,000×; (**d**) TEM micrography at 500,000×.

The AuNCs were synthesized by reduction of tetrachloroauric acid trihydrate with sodium borohydride and stabilized by 23-mercapto-3,6,9,12-tetraoxatricosan-1-ol using standard procedures [31]. They were characterized by ^1^H-NMR spectroscopy, transmission electron microscopy (TEM), zeta potential, and electrospray mass ionization. The resulting structure was Au_10_(SR)_15_^2-^ with chemical composition Au_10_C_285_H_585_O_75_S_15_ (Figure 1; see also Figures S1–S3 in the Supplementary Materials for details). The nanoclusters show a diameter of 1.5 ± 0.5 nm and a zeta potential of -2 mV.

The AuNCs were further functionalized with PpIX (AuNC-PpIX), DOX (AuNC-DOX), or both (AuNC-PpIX-DOX), using standard procedures (see Supplementary Materials). DOX was attached to the AuNc through (Z)-3,3′-(ethene-1,2-diylbis(sulfanediyl))dipropanoic acid, a ^1^O_2_-cleavable linker and we checked that it could be released by ^1^O_2_ (see Supplementary Materials, Figures S5 and S6).

### 3.2. AuNCs-PpIX-DOX Design Achieves a Synergistic Effect of Photo- and Chemotherapy in the Inactivation of Human Tumoral Cells

Dark and phototoxicity (after 2.4 J/cm^2^ of red irradiation) of all treatments was assessed by the MTT cytotoxicity assay on HeLa cells 48 h after treatments. The MTT assay is widely used for the assessment of cytotoxicity, cell viability, and cell proliferation studies [32].

**Figure 2:**
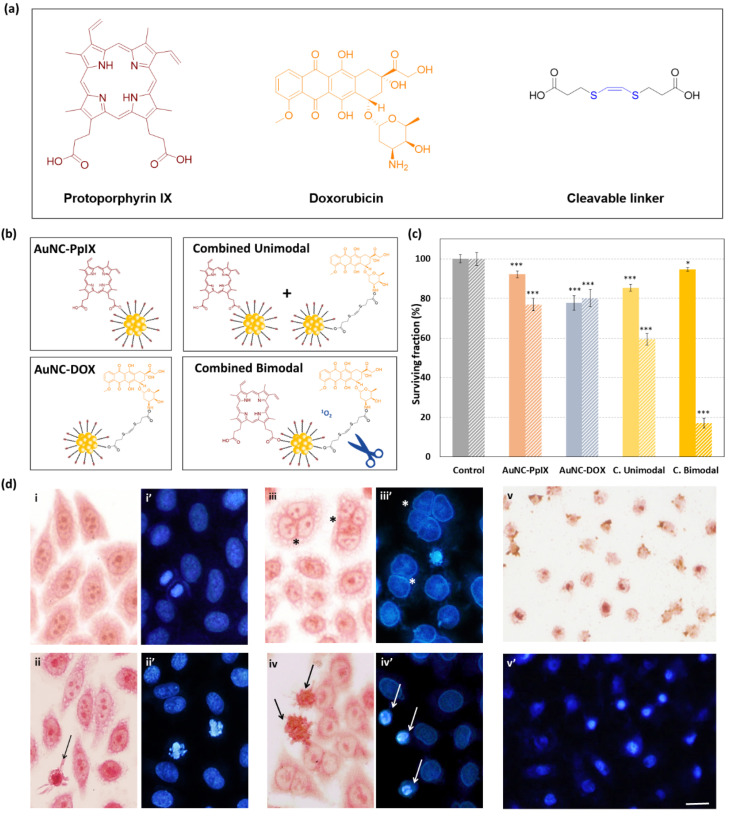
AuNC-PpIX-DOX treatment synergistically enhances cell death of HeLa cells. (**a**) Scheme of compounds used in different treatments; (**b**) Nanoconjugates and treatments; (**c**) MTT viability assay 48h after individual and combined treatments in dark conditions (plain bars) and after 2.4 J/cm^2^ of exposure to red light (striped bars). Data correspond to mean ± S.D. values from at least three independent experiments. Statistically significant differences are labeled as * *p* < 0.01 and *** *p* < 0.001; (**d**) Neutral red (**i**–**v**) and H-33258 (**i’**–**v’**) staining on HeLa cells 48 h after treatments. (**i**,**i’**) Control cells, (**ii**,**ii’**) AuNC-PpIX treatment, (**iii**,**iii’**) AuNC-DOX treatment, (**iv**,**iv’**) combined unimodal treatment, and (**v**,**v**’) combined bimodal treatment. Multinucleated cells (*) and cells with apoptotic morphology (arrows) were found. Scale bar: 10 μm in all images.

This assay demonstrated that the combined bimodal treatment (AuNC-PpIX-DOX) induces synergistic toxicity, achieving over 80% of HeLa cell death (vs. 23% and 20% of toxicity with AuNC-PpIX and AuNC-DOX individual treatments, respectively). Moreover, cytotoxicity was drastically reduced when PpIX and DOX were attached to different AuNCs (combined unimodal treatment; Figure 2b). An explanation for this observation can be found in the PDT mechanism of action. Upon irradiation, PpIX reacts with cellular oxygen molecules leading to the formation of ROS, which cause photodamage to cellular components [6]. In our combined bimodal system, ^1^O_2_ also triggers the release of DOX molecules attached to AuNCs. If PpIX and DOX are attached to different AuNCs, ^1^O_2_ produced on one AuNC is very unlikely to trigger the release of DOX from another AuNC due to its short lifetime that prevents diffusion over long distances [33]. This would lead to a decrease in cytotoxicity of treatment, which is consistent with our MTT results.

MTT assay also showed that attachment to AuNCs reduces the cytotoxicity of individual drugs (when compared with free PpIX and DOX. See Supplementary Materials, Figure S4). This effect is especially remarkable with DOX (100% vs. 20 ± 4% of cell death in free vs. AuNC-attached DOX, respectively). Although at first sight this might look like a dissatisfying result, it also suggests that delivering DOX through AuNCs increases the safety profile of this drug, potentially decreasing its severe side effects, such as cardiomyopathy [34,35], as its toxicity would be limited to irradiated area. According to this hypothesis, after treatment with AuNC-DOX, DOX should be localized in the cytosol, as its attachment to AuNC would be retaining DOX from migrating into nuclei, where it intercalates into DNA inducing double-strand breaks [36].

Finally, it is important to note that the AuNC-PpIX-DOX nanocluster conjugate has almost null dark toxicity (5.0 ± 0.9% of cell death), which is one of the requirements for an ideal photodynamic treatment [37].

To further assess the cellular responses of HeLa cells after each treatment, we used NR (neutral red) for general cell morphology and Hoechst-33258 (H-33258) staining for DNA visualization (Figure 2c). Morphological alterations are a widespread and effective method to identify cellular responses and cell death mechanisms after a certain treatment [38,39,40].

Morphology of control cells was very similar to the cells treated with AuNCs-PpIX, although the presence of a small percentage of cells with apoptotic morphology was detected (arrow in Figure 2c ii). After AuNCs-DOX treatment we detected an important increase in the number of multinucleated cells (* in Figure 2c iii,iii’), but the presence of dead cells was insignificant. Alterations on cell morphology were significantly increased after combined treatments. After the combined unimodal treatment, we were able to detect cells with early apoptotic morphology (cell shrinkage and pyknosis) even 48 h after treatment indicating that an important percentage of cells were progressively undergoing apoptosis (Figure 2c iv,iv’). The combined bimodal treatment was even more cytotoxic, and most cells had already undergone cell death 48 h after the treatment when only cytoplasm and nuclei remnants could be found still attached to the culture substrate (Figure 2c v,v’).

Synergistic effects between photo- and chemo-therapy has been previously reported in several clinical and preclinical studies [20,25,41,42,43,44,45]. However, to our knowledge, this is the first time that AuNCs are used as a platform for a light-controlled combination cancer therapy with PpIX and DOX.

### 3.3. AuNC-PpIX-DOX Treatment Triggers Massive Apoptotic Cell Death

**Figure 3:**
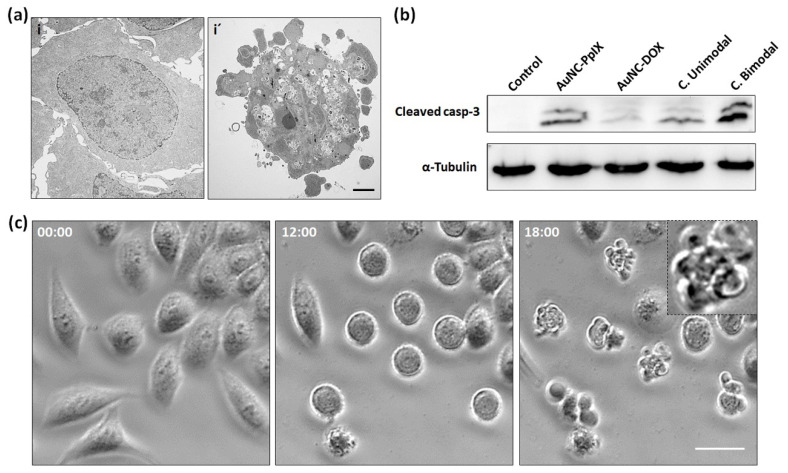
Combined bimodal treatment triggered apoptotic cell death of HeLa cells. (**a**) TEM micrographs of a control cell (**i**) and a cell 24 h after combined bimodal treatment (**i’**). Scale bar: 2 μm; (**b**) Caspase-3 cleavage revealed by western blotting; (**c**) Selected images of time-lapse video from the same field of a HeLa cells after combined bimodal treatment. Numbers at the top-left of each frame denote the time elapsed from the moment of irradiation (0, 12, and 18 h). Scale bar: 20 μm.

TEM microscopy examination confirmed the onset of apoptosis features. Interphase control cells (Figure 3a-i) showed a polygonal shape and large size nuclei with lax chromatin and prominent nucleolus. 24 h after combined bimodal treatment, cells showed evident morphological alterations such as chromatin condensation, cellular shrinkage, and extensive plasma membrane blebbing (Figure 3a-i’), which are consistent with typical apoptotic morphology [38].

Despite its value, morphological criteria may not be sufficient to clearly establish the type of cell death and thus, biochemical data should be provided for a more solid conclusion [40].

For this reason, we performed western blotting of cleaved caspase-3, an effector caspase that is proteolytically activated during apoptosis and plays a central role in the apoptotic pathway [46]. As shown in Figure 3b, we were able to detect caspase-3 activation after AuNCs-PpIX and combined unimodal treatment, but its activation was remarkably higher in cells treated with combined bimodal treatment.

Finally, time-lapse video microscopy during 24 h after combined bimodal treatment showed that this treatment induces a progressive cell rounding that finally ends in cell death with apoptotic features 18 h after treatment (Figure 3c. See Supplementary Materials for a complete video of control and treated cells). This unequivocally confirms that the combined bimodal treatment with AuNC-DOX-PpIX can induce cell death of human tumoral cells through apoptosis.

### 3.4. AuNC-PpIX-DOX Complexes are Internalized through Macropinocytosis Mechanism

To determine the internalization mechanism of AuNC-PpIX-DOX into cells, HeLa cells were incubated for 18 h with these nanoconjugates and were subsequently fixed for TEM microscopy (see Materials and Methods section). Compared to other metallic nanoparticles, gold nanoclusters are stable under the electron beam and provide excellent contrast in TEM. Thus, TEM is considered the best method to observe individual gold nanoparticles inside the cellular compartments [47].

**Figure 4:**
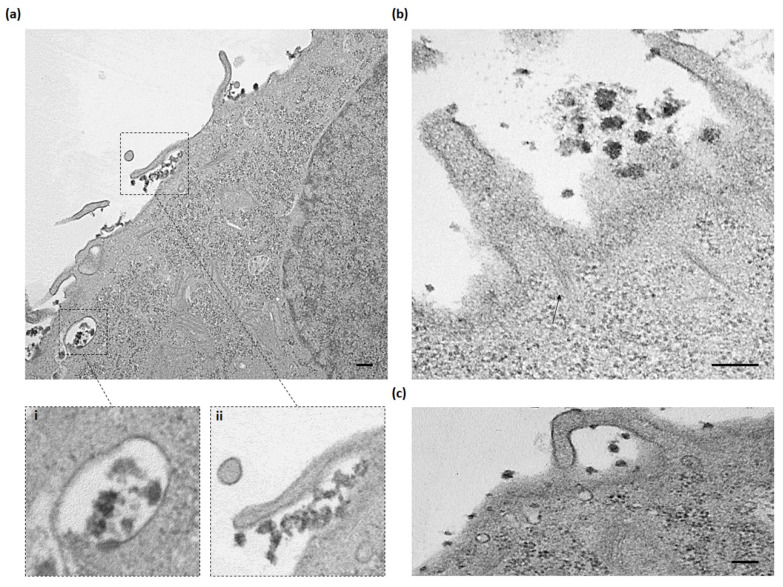
AuNC-PpIX-DOX complexes are internalized through a macropinocytic mechanism. (**a**–**c**) TEM micrographs showing AuNC-PpIX-DOX internalized into HeLa cells mediated by actin filaments (arrow). Scale bar: 0.2 μm.

TEM micrographs showed that AuNC-PpIX-DOX nanoconjugates were mainly internalized through macropinocytosis. Images collected in Figure 3 showed the main morphological features associated with this endocytic mechanism: extensive plasma membrane reorganization or ruffling (Figure 4a-ii,b,c) and subsequent formation of an external macropinocytic structure that is then enclosed and internalized (Figure 4a-i) [48]. TEM high resolutions even allowed us to visualize actin filaments involved in the formation of membrane ruffles (arrow in Figure 4b). Previous evidence on the cellular entry of gold nanoparticles shows that macropinocytosis is one of the major internalization mechanisms for this type of nanostructures [49,50,51].

Macropinocytosis is an endocytic pathway driven by actin microfilaments that allow the incorporation of extracellular material through large endocytic vesicles, named macropinosomes. Recently, micropinocytosis has been identified as a mechanism by which cancer cells are supplied with nutrients to support their metabolic needs and sustain cancer cell proliferation. It has been described that this endocytic mechanism is up-regulated in certain types of cancer and there is evidence that macropinocytosis level is directly related to tumor growth [52,53,54].

According to this, the fact that our AuNC-PpIX-DOX complexes are internalized by cells through macropinocytosis could contribute to its preferential accumulation into tumor cells, thus reducing systemic toxicity and subsequent side effects.

### 3.5. Attachment to AuNCs Increases PpIX Accumulation inside Tumor Cells

**Figure 5:**
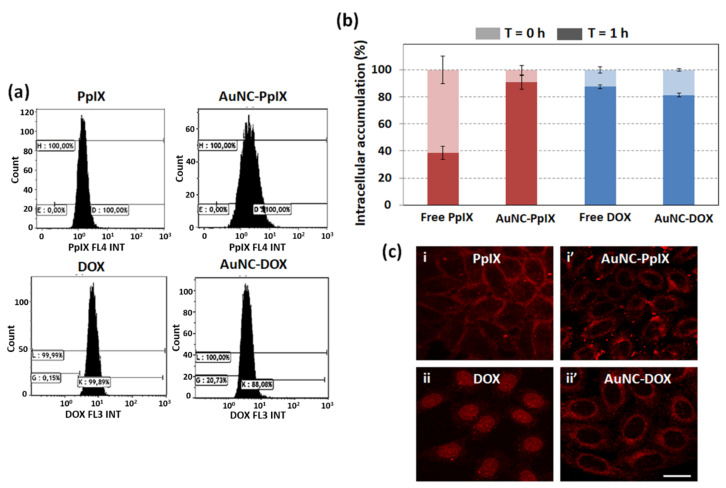
Attachment to AuNCs increases PpIX accumulation inside HeLa cells. (**a**) Analysis of free and AuNCs-attached PpIX and DOX accumulation by flow cytometry; (**b**) Relative accumulation of compounds inside HeLa cells just after washing and 1 h later; (**c**) Subcellular localization of free and AuNCs- attached PpIX and DOX. Scale bar: 20 μm.

To analyze if attachment to AuNCs was affecting drug accumulation into HeLa cells, we used flow cytometry to measure the amount of PpIX and DOX inside HeLa cells (Figure 5). We observed a significant increase in PpIX accumulation due to its attachment to AuNCs (Figure 5a). This effect was not detected in DOX accumulation. Furthermore, we measured drug accumulation just after washing and one hour later (Figure 5b). This experiment allowed us to assess that up to 60% of free PpIX are expelled from cells within one hour, while this percentage is reduced to less than 10% when PpIX is delivered using AuNCs. Once again, we did not detect any efflux with free DOX, likely due to its fast diffusion into the cell nucleus where it intercalates to DNA (see below).

Even though we have detected an increase in PpIX accumulation inside cells when it is attached to AuNCs, MTT assay showed that attachment to AuNCs reduces its phototoxicity (see Supplementary Materials Figure S4 vs. Figure 2a). These two results, which may seem contradictory, became meaningful when we analyzed drug localization at the subcellular level. As can be seen in Figure 5c, free PpIX was mainly accumulated near the plasma membrane, while AuNC-PpIX seems to be more diffuse in the cytosol. Membrane localization has been related to higher PDT efficacy. Thus, even mild PDT-induced membrane oxidation can produce alterations of essential membrane functions, which normally lead to cell death [15]. This is consistent with our results as, despite its lower accumulation inside cells, free PpIX is triggering higher phototoxicity than AuNC-PpIX due to its membrane localization.

In turn, confocal microscopy also revealed that free DOX is accumulated inside HeLa cells nuclei (Figure 5c-ii) while AuNC-DOX is retained in the cytosol since no fluorescence could be detected inside the cell nucleus (Figure 5c-ii’).

### 3.6. Attachment of DOX to AuNCs Prevents DOX-Induced Double-Strand Breaks in HeLa Nuclei

**Figure 6:**
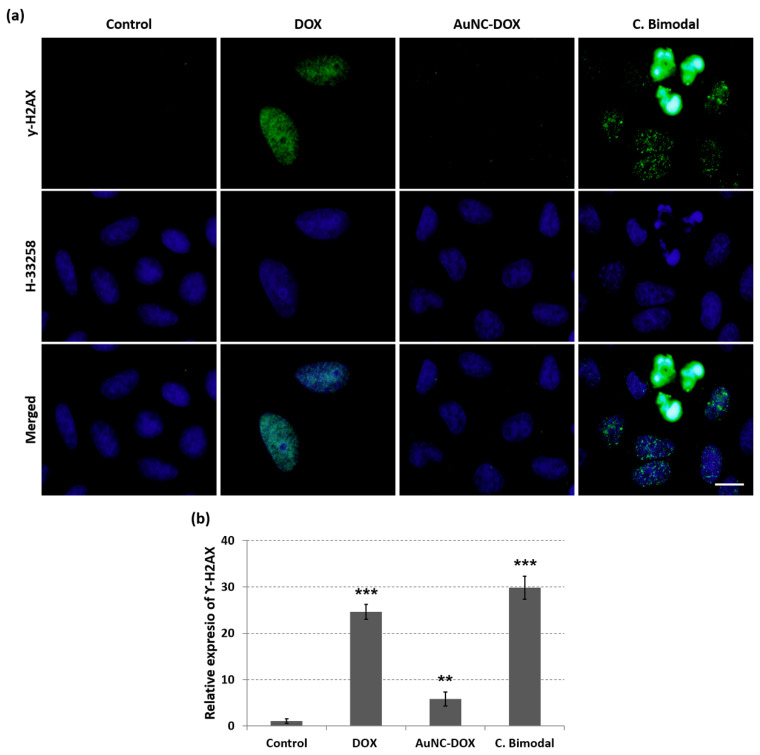
Attachment of DOX to AuNCs prevents DOX-induced double-strand breaks in HeLa nuclei. (**a**) Immunofluorescence of histone ƴ-H2AX (green) and H-33258 staining of DNA (blue) in untreated (control) cells and cells treated with DOX, AuNC-DOX, or combined bimodal treatment. Scale bar: 10 μm; (**b**) Quantification of relative expression of histone ƴ-H2AX on HeLa cells after each treatment. Statistically significant differences are labeled as ** *p* < 0.005, and *** *p* < 0.001.

DOX is a common chemotherapeutic agent used in the treatment of a variety of cancer types. Its mechanism of action is based on its intercalation into DNA and stabilization of topoisomerase II complex, thus inducing double-strand breaks in the DNA chain and preventing DNA double helix from being repaired [36]. To analyze the DOX effect on HeLa cells, we performed indirect immunofluorescence for histone ƴ-H2AX (Figure 6a) after 3 h of incubation with free DOX or 3 h after irradiation of AuNC-DOX or bimodal AuNC-PpIX-DOX treated cells. This histone is involved in DNA repair and it is considered to be a biomarker for DNA double-strand breaks [55]. After this assay, we were able to detect a high signal for histone ƴ-H2AX in DOX treated cells, but no signal was detected after AuNC-DOX treatment, corroborating that attachment of DOX to AuNCs is preventing DOX entry into HeLa nuclei. However, strong histone ƴ-H2AX signal in HeLa nuclei was detected after the combined bimodal treatment. This result indicates that ^1^O_2_ produced after activation of PpIX by red light can cleave the DOX linker to AuNCs. The released DOX is then internalized into the nucleus inducing DNA double-strand breaks. Using Fiji software, we have quantified the relative expression of histone ƴ-H2AX on HeLa cells (Figure 6b) after each treatment. Attachment of DOX to AuNCs through a ^1^O_2_-cleavable linker completely arrests its cytotoxic action in the absence of a PS and light.

## 4. Conclusions

We have successfully prepared and characterized a new photopharmacological nanosystem based on AuNCs with attached PpIX and DOX. Treatments of HeLa cells using AuNC-PpIX-DOX show cytotoxic synergistic chemo- and photodynamic effect, due to both the damage inflicted by DOX and ^1^O_2_ released in a controlled way by light activation of PpIX. A strong reduction of DOX cytotoxicity is also observed relative to the free drug, suggesting that the nanosystem could decrease the side effects associated with this drug. In summary, light-triggered co-delivery of ^1^O_2_ and DOX shows strong potential for spatiotemporal control of cancer therapy.

## Figures and Tables

**Table 1:** Concentration of gold nanoclusters and drugs (Protoporphyrin IX, PpIX, and Doxorubicin, DOX) present in stock solutions and hydrodynamic diameters and zeta potentials of all samples.

Samples	[PpIX] (mM)	[DOX] (mM)	[AuNC] (mg/mL)	Diameter [nm]	Zeta Potential [mV]
AuNCs	-	-	15	1.5 ± 0.5	−2
AuNC-PpIX	2.5	-	15	1.5 ± 0.5	−5
AuNC-DOX	-	0.75	15	1.5 ± 0.5	−2
AuNC-PpIX-DOXx	1.3	0.8	15	1.5 ± 0.5	−3

## Supplementary Materials

The following are available online at https://www.mdpi.com/2079-4991/10/12/2474/s1, Synthesis of undecyl-tetraethyleneglycol ligands, Synthesis of the singlet oxygen-cleavable linker (Z)-3,3’-(ethene-1,2-diylbis(sulfanediyl))dipropanoic acid, Synthesis of gold nanoclusters, Conjugation of Protoporphyrin IX and Doxorubicin to the gold nanoclusters, Individual and combined treatment with free Protoporphyrin IX and Doxorubicin in HeLa cells, Time-lapse video microscopy of HeLa cells after Combined Bimodal treatment, Singlet oxygen-induced release of Doxorubicin from AuNC-DOX nanoconjugates, Reactivity of singlet oxygen with the cleavable linker Video S1: Control HeLa cells, Video S2—Combined bimodal treated HeLa cells. Figure S1: NMR spectra of –OH terminated tetraethyleneglycol undecyl-thiol ligands (red) and AuNCs (blue)., Figure S2: ESI mass spectrum of AuNCs, Figure S3: (A) Experimental mass spectrum of gold nanoclusters; (B) theoretical mass spectrum corresponding to Au_10_(SR)_15_^2-^ nanocluster, with chemical composition Au_10_C_285_H_585_O_75_S_15_; (C) theoretical mass spectrum corresponding to Au_10_(SR)_13_^2-^ nanocluster, with chemical composition Au_10_C_247_H_507_O_65_S_13_ and (D) theoretical mass spectrum corresponding to Au_10_(SR)_11_^2-^ nanocluster, with chemical composition Au_10_C_209_H_429_O_55_S_11_, Figure S4: MTT viability assay 48 h after individual and combined treatments with free drugs in dark conditions (plain bars) and after 2.4 J/cm^2^ of red light (striped bars). Data correspond to mean ± S.D. values from at least six different experiments. Statistically-significant differences are labelled as *** *p* < 0.001, n.s. non-significant, Figure S5: Singlet oxygen-induced release of Doxorubicin from Gold nanocluster-doxorubicin conjugates bound through a singlet oxygen cleavable linker. As controls, the fluorescence of the non-functionalized nanoclusters and of free doxorubicin was recorded under the same conditions, Figure S6: Rate constant for the reaction of singlet oxygen with the linker. The rate of singlet oxygen disappearance increased linearly with the concentration of the linker.
